# Asylum Superintendents Appointed

**Published:** 1891-01

**Authors:** 


### ASYLUM SUPERINTENDENTS APPOINTED.

Governor Hogg, who was inaugurated on the 20th inst., as early as Jan. 5th made it known that Dr. John Preston, late 1st assistant physician at Austin, and a prominent candidate for Superintendent of the State Lunatic Asylum, at Austin, would be assigned as Superintendent of the asylum at Terrell, vice Dr. Wallace, whose term expires with the change of administration. When the appointment was tendered Dr. Preston, (who, it will be remembered, recently resigned his position as first assistant at the asylum and removed to San Antonio), the Doctor was quite surprised, and replied that he had not asked for the Terrell asylum and had never given the subject a thought. He also said that he had too much regard for Dr. Wallace the long-time Superintendent, the father of that institution, in fact, to antagonize him, and therefore asked a week's time in which to consider the Governor's offer, even after the Governor assured him that he did not intend to re-appoint Dr. Wallace. At the end of the week Dr. Preston accepted the appointment. We are pleased to learn that he has tendered the position of first assistant to Dr. F. S. White, the present incumbent, and who has been Dr. Wallace's chief-of-staff from the founding of the institution.

It has been announced also, that the Governor has appointed, Dr. W. W. Reeves, of Wills Point, VanZandt county, to the Suintendency of the Austin Insane Asylum.

Dr. Reeves is a prominent and very popular physician of North-east Texas; a member of the Texas State Medical Association, and will doubtless make an excellent officer. The JOURNAL has no information as to the extent of his experience in the management of an asylum or the treatment of the insane; and, it is generally understood, that experience is a prime requisite stipulated by the law, a *sine qua non* to the appointment; but the Governor, who is ex-Attorney-General, (and who better qualified to expound or interput the law?) construes the section refered to, to mean experience in the practice of medicine, only. Under this construction *any* physician who has ever visited a sick man, could come in, supposing him to possess the *other* requisites. (We will not name them). The JOURNAL has no fault to find with Dr. Reeves' appointment, though he was not our choice; we believe he will reflect honor on the office, and we beg hereby to extend to him our congratulations.

The appropriateness of Dr. Preston's appointment at the head of one of the great asylums of the State, is all but universally acknowledged, though his friends would much prefer the reversal of the appointments, Dr. Preston at Austin, and Dr. Reeves at Terrell, in his own county. This is something like the policy of our generals during the war, one which we, a private, could not understand or appreciate, *i. e.*, to send Mississippi troops to Virginia, and Virginia troops to Texas! The inmates of the Terrell asylum, who were greatly attached to Dr. Wallace, may be consoled by the assurance that in Dr. Preston they will find a worthy successor to their old favorite. We believe no fault was found of Dr. Wallace; this is only another exemplification of the well-understood and accepted maxim in politics, that "to the victor belongs the spoils." Wallace's only offense, so far as we know, was that he was not a "Hogg man"; he tossed not high his ready cap in air, nor lifted his voice in the convention, for the great popular favorite, our excellent new Governor.

